# Case Report: Dangerous liaisons between splenectomy and eltrombopag-induced chronic thromboembolic pulmonary hypertension in patients with immune thrombocytopenia: report of two cases and review of the literature

**DOI:** 10.3389/fcvm.2025.1508574

**Published:** 2025-02-12

**Authors:** Roberto Castelli, Enrico Atzori, Alessandro Palmerio Delitala, Salvatore Antonio Masala, Valentina Micheluzzi, Enrico Ponti, Giuseppe Sanna, Dante Castro, Antonio Gidaro, Mattia Donadoni, Roberto Manetti, Pierluigi Merella, Nicia Isabella Profili, Andrea Maria D'Armini, Gavino Casu

**Affiliations:** ^1^ Department of Medicine, Surgery and Pharmacy, University of Sassari, Sassari, Italy; ^2^ Clinical and Interventional Cardiology, University Hospital, Sassari, Italy; ^3^ Department of Biomedical and Clinical Sciences Luigi Sacco, Luigi Sacco Hospital, University of Milan, Milan, Italy; ^4^ Cardiac Surgery, University of Pavia, Fondazione IRCCS Policlinico San Matteo, Pavia, Italy

**Keywords:** primary immune thrombocytopenia, thrombopoietin receptors agonists, eltrombopag, splenectomy, chronic thromboembolic pulmonary hypertension, pulmonary thromboendarterectomy

## Abstract

**Introduction:**

Primary immune thrombocytopenia is an autoimmune bleeding disorder characterized by variable immune-mediated platelet destruction. These patients have reported thrombotic complications, both venous and arterial, in addition to bleeding. Splenectomy and thrombopoietin receptor agonists are recommended for patients who do not respond to steroids or immunosuppressive treatments. Chronic thromboembolic pulmonary hypertension is a rare disease that results from a persistent, organized thromboembolic obstruction of the pulmonary arteries due to an incompletely resolved pulmonary embolism.

**Case presentations:**

We report two cases of chronic thromboembolic pulmonary hypertension induced by a thrombotic mechanism after treatment with splenectomy and Eltrombopag, a thrombopoietin receptor agonist, for refractory primary immune thrombocytopenia. Consequently, the patients were referred for surgical pulmonary thromboendarterectomy therapy as suggested.

**Conclusion:**

In older patients, those with a history of thrombotic manifestations, or those with high-risk factors, clinicians should evaluate and monitor the risk of thrombotic events and chronic thromboembolic pulmonary hypertension when treating primary immune thrombocytopenia with splenectomy and Eltrombopag.

## Introduction

Primary immune thrombocytopenia (ITP) is an autoimmune bleeding disorder characterized by variable immune-mediated platelet destruction due to autoantibodies primarily directed against Gp Ib/IX and Gp IIb/IIIa, as well as impaired platelet production (1, 2). With the advent of routine complete blood counts, the annual incidence of ITP among adults is estimated to be between 1 and 6 cases per 100,000. ITP leads to bleeding in 60% of patients. Bleeding manifestations range from mild skin bruises to life-threatening hemorrhages. Severe bleeding complications occur in only 6% of patients, and cerebral hemorrhage develops in just 0.4% of patients with ITP (2, 3).

Severe bleeding occurs when the platelet count is below 30 k/*μ*l. However, other factors, such as the duration of thrombocytopenia, previous hemorrhagic manifestations, and the concomitant use of drugs affecting hemostasis, should also be considered when evaluating hemorrhagic risk in ITP patients. About 60%–70% of adults develop a chronic condition lasting more than 12 months, defined as chronic ITP (cITP) (3–5). ITP is a chronic disease in adults, meaning its prevalence significantly exceeds its incidence. According to a database review conducted by the French National Health Insurance System, the incidence of ITP cases requiring chronic therapy and/or hospitalization was 2.9 per 100,000 person-years. The study found that individuals over 60 had the highest incidence rate, with men over 75 experiencing 9 cases per 100,000 person-years (6).

For individuals with ITP, determining the need for therapy to increase platelet count involves a quick clinical evaluation that considers several factors, such as the presence, site, acuteness, and severity of bleeding, as well as platelet count, prior treatments, and bleeding risk factors. Treatment is essential for those experiencing critical or severe bleeding and requires immediate attention, which may include platelet transfusion, intravenous immune globulin (IVIG), glucocorticoids, and other treatments as needed. Despite thrombocytopenia, close monitoring and attention to other bleeding risk factors may be sufficient for individuals with minor or no bleeding.

If the platelet count is less than 20 k/*μ*l, therapy to increase platelet count is generally appropriate, except for rare cases where patients have antiplatelet antibodies that impede platelet function. Managing these patients is complex and requires the intervention of a hemostasis expert (1).

Glucocorticoids and IVIG are two treatments that can increase platelet count. However, they differ in their mechanisms of action, speed of platelet count increase, side effects, and costs.

The rate of platelet count increase is faster with IVIG compared to glucocorticoids. Specifically, on the fifth day of treatment, the platelet count exceeded 50 k/*μ*l in 79 percent of patients receiving IVIG compared to 60 percent receiving steroids. While most patients respond to glucocorticoids within two to five days, it may take up to two weeks for some individuals (1).

&For individuals who do not achieve a stable and safe platelet count with first-line therapy, alternative approaches such as multi-agent combinations, second-line agents, or splenectomy can be considered (1).

Thrombopoietin receptor agonists (TPO-RAs) are indicated in patients who are unresponsive or refractories to steroids or immunosuppressive therapies (7). The TPO-RAs currently approved for primary ITP are Romiplostim, Eltrombopag, and Avatrombopag, while Hetrombopag is a novel TPO-RA approved only in China to treat ITP. Eltrombopag is an oral agent approved for the management of chronic ITP in patients aged over 1 year, chronic hepatitis C with thrombocytopenia, and severe aplastic anemia that does not respond to immunosuppressive therapy. It works by binding to the TPO-R receptor in the bone marrow, thereby stimulating platelet production (8). Clinical studies have demonstrated that Eltrombopag increases platelet counts in 60%–80% of patients and helps reduce bleeding (9, 10). While common side effects include headache and nasopharyngitis, more serious adverse events have been reported, such as elevated liver enzymes, cataracts, thrombosis, and bone marrow fibrosis, which in some cases have led to treatment discontinuation (11).

Besides bleeding, thrombotic complications, both venous and arterial, have been reported in cITP, which is likely multifactorial due to both the thrombogenicity of ITP and an individual additional risk factor (12). Studies have shown evidence of dysregulated pro- and anti-inflammatory cytokines in ITP, suggesting that ITP is an inflammatory disease. This increased inflammatory activity can lead to thrombosis. Other factors inherent to ITP that may contribute to prothrombotic state include a high proportion of young, activated platelets and the presence of procoagulant, proinflammatory microparticles (13). While these factors are elevated in patients with ITP, there is no definitive evidence linking them to increased thrombosis.

Similar to the risk of bleeding, older age and the presence of comorbidities are also associated with an increased risk of thrombosis in ITP. According to one analysis, older age (>60), steroid use, splenectomy, and a combination of ≥3 vascular risk factors (including diabetes mellitus, hypercholesterolemia, arterial hypertension, smoking, atrial fibrillation, valvular disease, and coronary disease) were independent predictors of both venous and arterial thrombotic events. Additional risk factors for thromboembolic events include obesity, a postoperative state, male sex, and cancer (14).

Both first- and second-line treatments for immune thrombocytopenia (ITP) can increase the risk of thrombosis. For instance, intravenous immunoglobulin (IVIg) treatment may contribute to thrombosis by increasing blood viscosity and platelet activation (14).

CTEPH is a serious disease that significantly reduces both the quality and length of life. It is characterized by the presence of obstructive fibrotic thromboembolic material in the pulmonary vasculature and small vessel arteriopathy. Risk factors include various clinical conditions, including splenectomy (15). CTEPH is caused by persistent, organized thromboembolic obstruction of the pulmonary arteries due to an incompletely resolved pulmonary embolism. Pulmonary thromboendarterectomy (PTE) is the treatment of choice, and all patients should be evaluated for this surgical option. A large meta-analysis investigating the prevalence of splenectomy in CTEPH patients was recently published. The pooled crude prevalence of splenectomy in CTEPH patients was confirmed to be 4%, with individual studies reporting prevalence rates ranging from 2%–9% (16).

Here, we report two cases of CTEPH induced by a thrombotic mechanism following splenectomy and treatment with Eltrombopag for refractory ITP.

## Case presentation

### Case one

**Figure 1:**
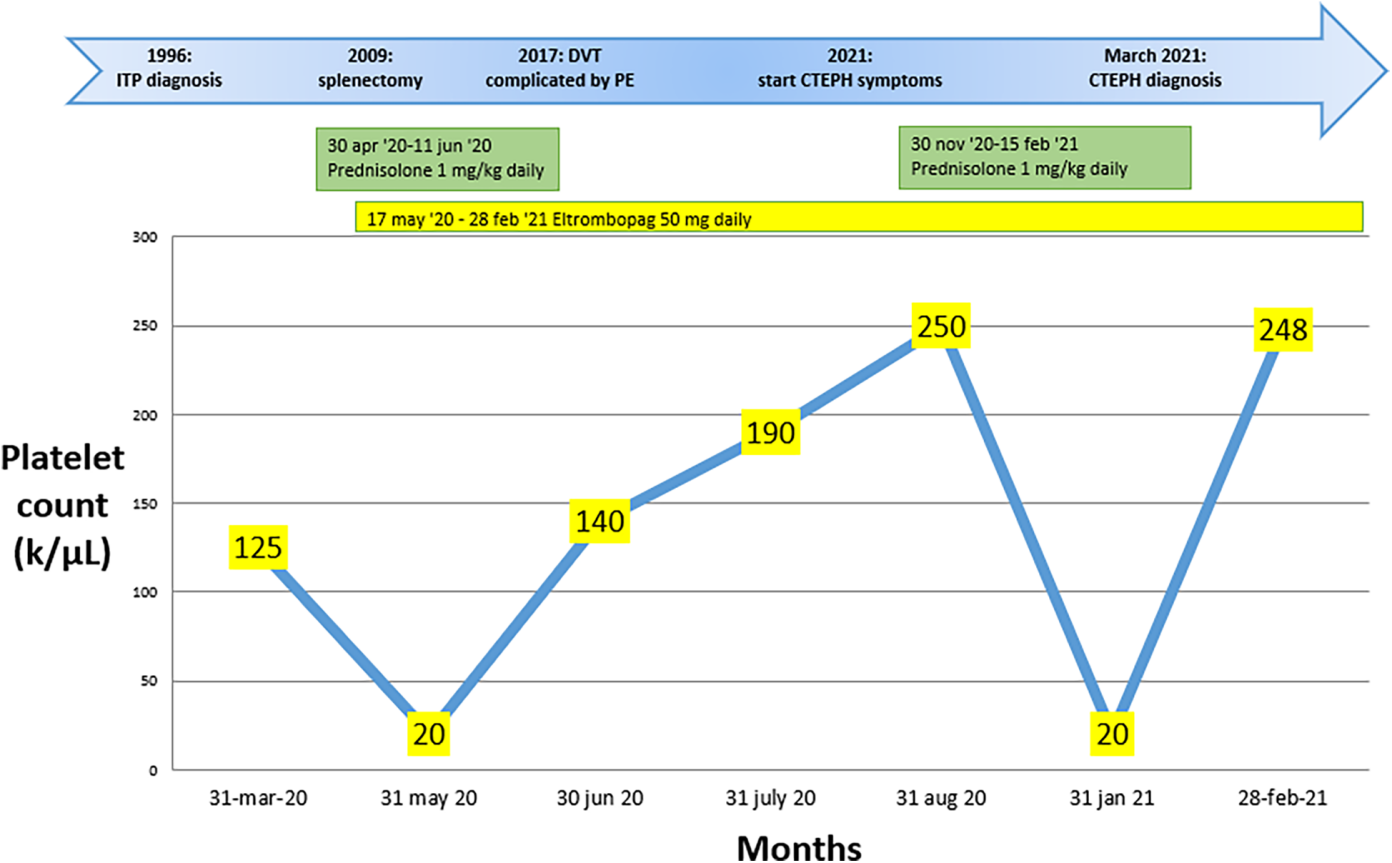
Changes in platelet count over the months during pharmacological treatment, along with the key steps that led to the diagnosis of chronic pulmonary embolism with thromboembolism in case 1.

A 68-year-old female with well-controlled hypertension, obesity, and a 25-year history of ITP came to our attention in February 2021. Initially, the patient had poorly controlled ITP despite corticosteroid treatment (Prednisone 1 mg/kg per day). Consequently, the patient underwent a splenectomy in 2009 without any benefit and only achieved an optimal platelet count starting from May 31, 2020, after beginning therapy with Eltrombopag 50 mg daily (17 May 2020). The platelet count throughout the months of treatment with steroids and Eltrombopag is depicted in Figure 1. The patient had a transient ischemic attack in 2019 and reported an episode of pulmonary embolism with bilateral segmental and sub-segmental involvement of the pulmonary arteries in November 2021. At the time of diagnosis, signs of pulmonary hypertension and right ventricular (RV) dysfunction were evident, with an RV diameter (RVD) of 45 mm and compromised ventricular function, as indicated by a TAPSE of 17 mm, a tricuspid regurgitation velocity (S′) of 9.5 cm/s, and an estimated pulmonary artery pressure (PAPS) of 82 mmHg. Additionally, left lower limb deep vein thrombosis was found, and anticoagulation with Rivaroxaban 15 mg twice daily was started. After three weeks, the Rivaroxaban dose was reduced to 20 mg once a day.

**Figure 2:**
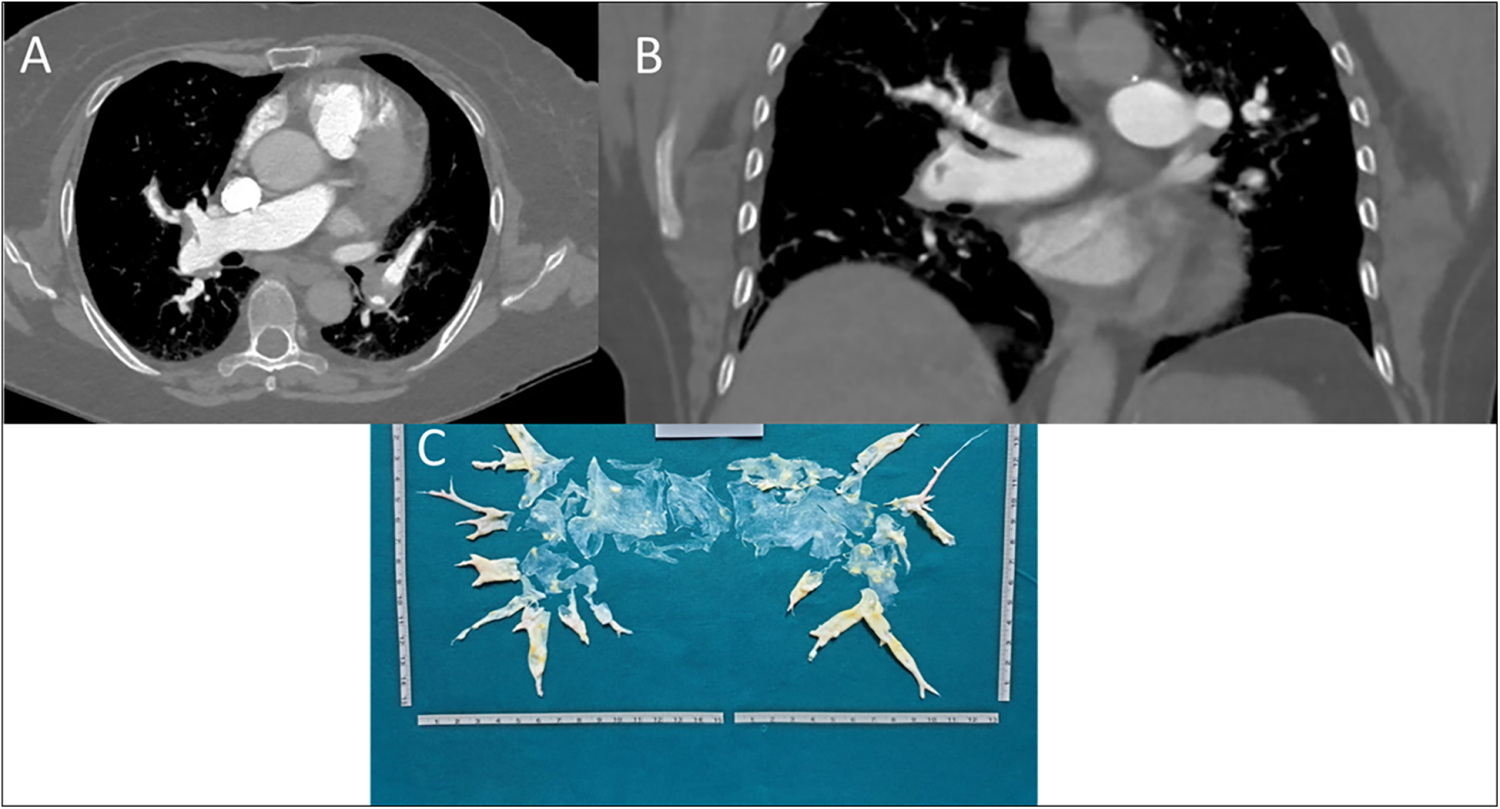
**(A)** axial scansion of chest CT with contrast. A filling defect of a thrombo-embolic nature in the right pulmonary artery, between the middle and lower lobe tributary branches, can be observed. The arrow also shows small thrombi in the left pulmonary artery branches. **(B)** Coronal scansion of chest CT with contrast. A filling defect of a thrombo-embolic nature in the branch of the right pulmonary artery tributary to the lower lobe can be observed. **(C)** Postoperatively surgical finding of pulmonary thromboendarterectomy.

In February 2021, due to worsening dyspnea over a few weeks, including symptoms at rest (Class IV of WHO functional classification), the patient was admitted to a hospital. Blood gas analysis revealed type 1 respiratory failure requiring oxygen therapy. The complete blood count, including a platelet count of 248 k/*μ*l, and the D-dimer level (688 ng/ml) were within normal limits. However, the pro-brain natriuretic peptide (pro-BNP) was mildly elevated at 881 pg/ml. Echocardiographic findings suggested a high probability of pulmonary hypertension, and axial computed tomography revealed partial filling defects in the segmental and sub-segmental branches of the pulmonary arteries, consistent with CTEPH (Figures 2A,B). A ventilation/perfusion (V/Q) scan and right heart catheterization (RHC) were performed, showing bilateral ventilation-perfusion mismatch and pre-capillary pulmonary hypertension with a severe reduction of cardiac index (CI) [mean pulmonary arterial pressure (mPAP) 41 mmHg, Pulmonary Artery Wedge Pressure (PAWP) 12 mmHg, pulmonary vascular resistance (PVR) 8 WU, CI 1.7 L/min/m^2^]. Histological examination of the thrombus confirmed that it was composed of platelets, collagen, and fibrin, having undergone reorganization over time. Therapeutic measures included discontinuation of Eltrombopag, increased corticosteroid dosage, and replacement of Rivaroxaban with Fondaparinux 10 mg daily (patient weight over 100 Kg). The patient was referred for PTE at the Cardiac Surgery Center (Figure 2C). In the immediate pre-operative period, intravenous immunoglobulins were administered due to the significant reduction of platelets. Given the thrombophilic diathesis, a caval filter was placed during the procedure, and anticoagulation therapy with vitamin K antagonists was initiated. At the end of the intervention, an immediate and pronounced decrease in mPAP values was observed. This finding was confirmed by pre-discharge right heart catheterization (RHC), which showed a 47% reduction in mPAP, a 71% reduction in PVR and a 38% increase in cardiac output. Finally, the patient no longer required daytime oxygen therapy.

### Case two

A 68-year-old female patient with hypothyroidism, arterial hypertension, and a history of ITP since 2004 was initially treated with azathioprine and corticosteroids. Despite an ineffective splenectomy performed in 2014, the patient's condition was well-controlled for three years with Eltrombopag therapy.

**Figure 3:**
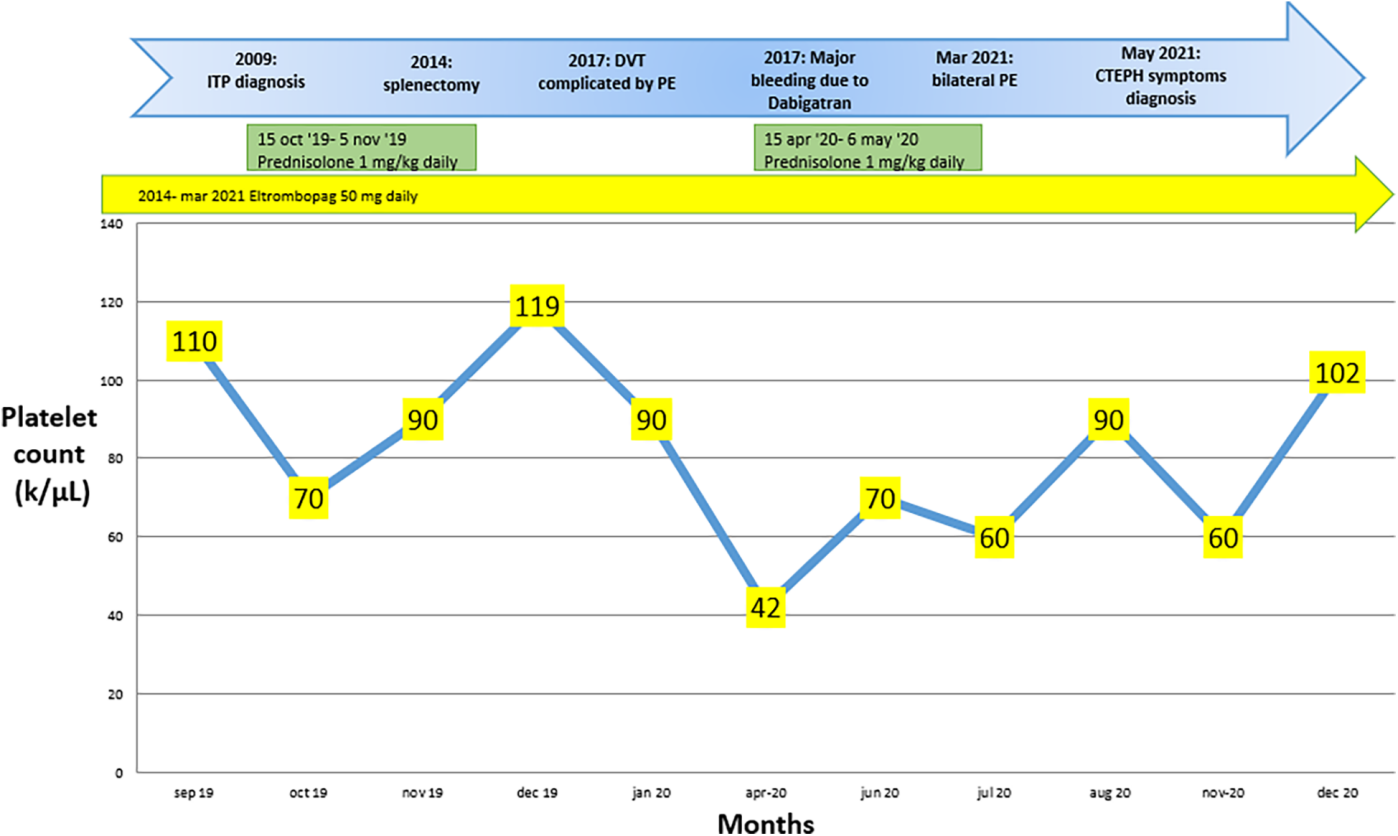
Changes in platelet count over the months during pharmacological treatment, along with the key steps that led to the diagnosis of chronic pulmonary embolism with thromboembolism in case 2.

In 2017, it was reported that a deep vein thrombosis in the left lower limb was complicated by pulmonary embolism. Dabigatran 150 mg twice daily was started and subsequently discontinued after six months due to major gingival bleeding. Platelet counts over the months during treatment with steroids and Eltrombopag are shown in Figure 3.

In March 2021, a new episode of bilateral pulmonary embolism occurred, with thrombotic filling defects in the lobar branches of pulmonary arteries, although no evidence of deep vein thrombosis in the lower limbs was found. At this point, Eltrombopag was discontinued, Rivaroxaban 15 mg twice daily was started, and the patient was discharged. After three weeks, the Rivaroxaban dose was reduced to 20 mg once daily.

In May 2021, the patient presented with mild exertional dyspnea (Class III of the WHO functional classification). Blood test revealed elevated pro-BNP levels (6,003 pg/ml), and EKG and echocardiography suggested right heart overload, consistent with CTEPH. Echocardiographic findings showed a right ventricular diameter (RVD) of 41 mm, with compromised right ventricular function, as indicated by a TAPSE of 18 mm and a tricuspid regurgitation velocity (S′) of 12 cm/s. The estimated pulmonary artery pressure (PAPS) was 58 mmHg.

**Figure 4:**
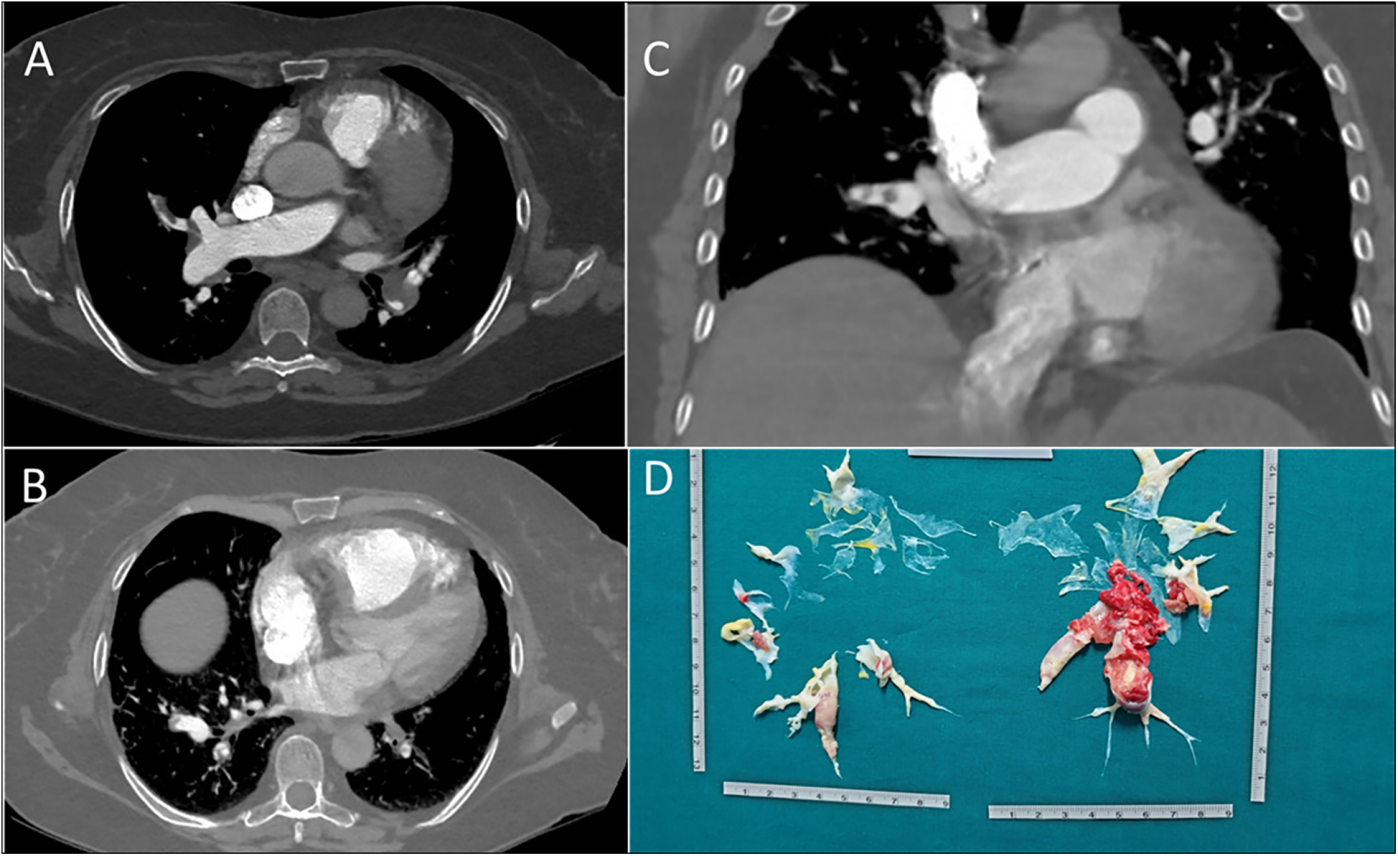
**(A)** axial scansion of chest CT with contrast. It shows a filling defect of a thrombo-embolic nature in the branch of the right pulmonary artery tributary to the middle lobe. **(B)** Axial scansion of chest CT with contrast. A filling defect of thrombo-embolic nature in the right and left pulmonary artery branches tributary to the lower lobes can be observed. **(C)** Coronal scansion of chest CT with contrast. It shows a filling defect of a thrombo-embolic nature in the branch of the right pulmonary artery tributary to the middle lobe. **(D)** Postoperative surgical finding of pulmonary thromboendarterectomy.

Axial computed tomography showed partial occlusion of pulmonary arteries, consistent with CTEPH (Figures 4A–C). The diagnosis was confirmed by a V/Q scan showing mismatch and by RHC, which revealed pre-capillary pulmonary hypertension and a severe reduction in CI (mPAP 54 mmHg, PAWP 10 mmHg, PVR 14 WU, CI 1.8 L/min/m^2^). Histological analysis of the thrombus revealed that it had undergone a process of reorganization over time, leading to the formation of a thrombus composed of platelets, fibrin, and collagen.

During the thrombophilia test, a partial reduction in protein C was observed. However, since the decrease was limited, it is not regarded as clinically significant. Corticosteroid therapy was initiated to address the progressive reduction in platelet count (to 25 k/*μ*l), and vitamin K antagonists were started for anticoagulation. The patient was referred to the PTE (Figure 4D). The surgical procedure was performed successfully; however, the patient unfortunately died during the postoperative period due to an infection.

## Discussion

cITP is considered a thrombotic condition rather than a hemorrhagic disorder. Several studies have shown that patients with cITP paradoxically have an increased risk of thrombotic complications, both venous and arterial, compared to the general population. The use of steroids and obesity in Case 1, and thyroid dysfunction and steroids in Case 2, along with splenectomy and Eltrombopag, may collectively increase the risk of blood clots. Steroids enhance platelet activity and elevate clotting factors such as fibrinogen, while obesity raises blood viscosity and promotes venous stasis, both of which increase the risk of thrombosis (17). Additionally, thyroid hormones influence coagulation: low levels induce a hypocoagulable and hyperfibrinolytic state, whereas high levels promote a prothrombotic state (18).

According to a Scandinavian cohort study involving 1,821 cITP patients, the risk of venous thrombosis (VTE) is 16.3 per 1,000 person-years [95% confidence interval (CI) 12.8–20.6] with 39 VTE events occurring in the ITP cohort during 4,233 person-years of follow-up [incidence rate (IR) = 9.2 per 1,000 person-years, 95% CI: 6.7–12.6] (6). Despite this, the literature lacks a connection between cITP and CTEPH (19).

With respect to a history of prior splenectomy, recent evidence suggests a relationship between this procedure and the development of CTEPH (20–22). Specifically, the study by Bollova et al. (2024) demonstrated that splenectomy can increase the risk of developing CTEPH, with a 5-year cumulative incidence of 3.2% compared to patients without a history of splenectomy. However, the study found that the strongest factor associated with the incidence of CTEPH after splenectomy was the presence of thrombophilia detected prior to screening echocardiography. Given these findings, and in agreement with our own cases, it is important to consider the potential role of Eltrombopag, in increasing this risk. Eltrombopag is used to treat various hematological conditions, including chronic thrombocytopenia. While it has been shown to be effective in increasing platelet levels, some studies suggest that Eltrombopag may influence the risk of thrombosis and potentially CTEPH. This could be due to Eltrombopag's procoagulant effects, which might exacerbate the already increased risk associated with splenectomy.

According with Hamed et al. (2023) the risk of CTEPH development is closely associated with the treatment used for cITP (23). In this study, Romiplostim, Rituximab, and Eltrombopag were associated with a significantly higher incidence of CTEPH compared to high dose-dexamethasone and prednisolone plus azathioprine (2.17%, 3.1%, and 6.25% vs. 12.3% and 20.9%, respectively; *p*-value < 0.001). This trial confirms that thrombotic factors are upregulated in response to corticosteroid exposure, as shown in *in vitro* studies. Furthermore, animal and population-based studies have suggested a higher risk of thromboembolic events with corticosteroid use (14).

While recent studies, suggest that the increased risk of thromboembolic events associated with TPO-RAs is not significant and is linked to specific patient characteristics, it is still essential to remain vigilant and collect more long-term data. Therefore, before considering the use of TPO-RAs, individual patient risk profiles should be evaluated. The lowest dose to maintain a safe platelet count (≥50 k/*μ*l) is recommended (24). For patients at high risk of thrombosis who are receiving TPO-RAs, anticoagulation or antiplatelet therapy can be considered once the platelet count has reached 50 k/*μ*l. The differences between our cases and those previously reported may arise from several contributing factors. While Eltrombopag effectively increases platelet counts, its procoagulant effects can heighten the risk of thrombosis, especially in patients with a history of splenectomy or thrombophilia. In Case 1, obesity further amplifies thrombosis risk by promoting venous stasis and increasing blood viscosity. In Case 2, thyroid dysfunction exacerbates coagulation imbalances. These factors, together with the procoagulant effect of Eltrombopag, could significantly increase the overall risk of thrombosis. This particular aspect, however, is not extensively discussed in the current literature, and further research is needed to better understand the potential risks associated with the use of Eltrombopag in such patients. It is important to note that the increased risk of CTEPH in patients treated with TPO-RAs may vary depending on individual characteristics, such as age, comorbidities, and prior thrombotic events. Thus, a personalized treatment approach and careful monitoring are crucial in managing these patients. Furthermore, while previous studies suggest a higher CTEPH risk with TPO-RAs, these findings are still evolving, and long-term follow-up studies are necessary to clarify the interaction between Eltrombopag, splenectomy, and the risk of CTEPH.

## Conclusion

To our knowledge, no case report of CTEPH requiring PTE after the use of TPO-RAs in cITP has been published in the literature. TPO-RAs have demonstrated high efficacy in increasing platelet count to >50 k/*μ*l in 60%–90% of patients with ITP. They should be offered as a second-line therapy, potentially with an increased, though not significant, risk of thrombosis. Nevertheless, in older patients, those with a history of thrombotic manifestations, and patients with high-risk factors (male sex, splenectomy, exposure to IVIg, systemic lupus erythematosus, or antiphospholipid syndrome), clinicians should evaluate and monitor the risk of thrombotic events and CTEPH when treating ITP patients with TPO-Ras, especially those with a history of splenectomy, to identify any early signs of CTEPH and to implement appropriate preventive measures. Further research is needed to clarify the potential impact of Eltrombopag on the risk of CTEPH and to develop clinical guidelines to effectively manage this risk.

## Data Availability

The original contributions presented in the study are included in the article/Supplementary Material, further inquiries can be directed to the corresponding author.

## References

[1] Cooper N Ghanima W. Immune thrombocytopenia. N Engl J Med. (2019) 381(10):945–55. 10.1056/NEJMcp1810479 31483965

[2] Castelli R Lambertenghi Delilliers G Gidaro A Cicardi M Bergamaschini L. Complement activation in patients with immune thrombocytopenic purpura according to phases of disease course. Clin Exp Immunol. (2020) 201(3):258–65. 10.1111/cei.13475 32515487 PMC7419927

[3] Piel-Julian M-L Mahévas M Germain J Languille L Comont T Lapeyre-Mestre M Risk factors for bleeding, including platelet count threshold, in newly diagnosed immune thrombocytopenia adults. J Thromb Haemost. (2018) 16(9):1830–42. 10.1111/jth.14227 29978544

[4] Castelli R Gidaro A Deliliers GL. Risk of thrombosis in elderly immune primary trombocytopenic patients treated with thrombopoietin receptors agonists. J Thromb Thrombolysis. (2020) 50(4):903–7. 10.1007/s11239-020-02083-x 32347510

[5] Ghanima W Cooper N Rodeghiero F Godeau B Bussel JB. Thrombopoietin receptor agonists: ten years later. Haematologica. (2019) 104(6):1112–23. 10.3324/haematol.2018.212845 31073079 PMC6545830

[6] Moulis G Palmaro A Montastruc J-L Godeau B Lapeyre-Mestre M Sailler L. Epidemiology of incident immune thrombocytopenia: a nationwide population-based study in France. Blood. (2014) 124(22):3308–15. 10.1182/blood-2014-05-578336 25305203

[7] Provan D Arnold DM Bussel JB Chong BH Cooper N Gernsheimer T Updated international consensus report on the investigation and management of primary immune thrombocytopenia. Blood Adv. (2019) 3(22):3780–817. 10.1182/bloodadvances.2019000812 31770441 PMC6880896

[8] Erickson-Miller CL Delorme E Tian SS Hopson CB Landis AJ Valoret EI Preclinical activity of eltrombopag (SB-497115), an oral, nonpeptide thrombopoietin receptor agonist. Stem Cells. (2009) 27(2):424–30. 10.1634/stemcells.2008-0366 19038790 PMC2729672

[9] Cheng G Saleh MN Marcher C Vasey S Mayer B Aivado M Eltrombopag for management of chronic immune thrombocytopenia (RAISE): a 6-month, randomised, phase 3 study. Lancet. (2011) 377(9763):393–402. 10.1016/S0140-6736(10)60959-2 20739054

[10] Bussel JB Provan D Shamsi T Cheng G Psaila B Kovaleva L Effect of eltrombopag on platelet counts and bleeding during treatment of chronic idiopathic thrombocytopenic purpura: a randomised, double-blind, placebo-controlled trial. Lancet. (2009) 373(9664):641–8. 10.1016/S0140-6736(09)60402-5 19231632

[11] Wong RSM Saleh MN Khelif A Salama A Portella MSO Burgess P Safety and efficacy of long-term treatment of chronic/persistent ITP with eltrombopag: final results of the EXTEND study. Blood. (2017) 130(23):2527–36. 10.1182/blood-2017-04-748707 29042367

[12] Nørgaard M Cetin K Maegbaek ML Kristensen NR Ghanima W Bahmanyar S Risk of arterial thrombotic and venous thromboembolic events in patients with primary chronic immune thrombocytopenia: a Scandinavian population-based cohort study. Br J Haematol. (2016) 174(4):639–42. 10.1111/bjh.13787 26456477

[13] Gidaro A Delitala AP Manetti R Caccia S Soloski MJ Lambertenghi Deliliers G Platelet microvesicles, inflammation, and coagulation markers: a pilot study. Hematol Rep. (2023) 15(4):684–95. 10.3390/hematolrep15040069 38132277 PMC10742513

[14] Lambert C Maitland H Ghanima W. Risk-based and individualised management of bleeding and thrombotic events in adults with primary immune thrombocytopenia (ITP). Eur J Haematol. (2024) 112(4):504–15. 10.1111/ejh.14154 38088207

[15] Ribas Sola J Sánchez-Corral Mena MÁ Riera-Mestre A. Update in the management of chronic thrombo-embolic pulmonary hypertension. Med Clin. (2024) 162(3):126–33. 10.1016/j.medcli.2023.08.006 37925273

[16] Zhang L Yan P Yang K Wu S Bai Y Zhu X Association between splenectomy and chronic thromboembolic pulmonary hypertension: a systematic review and meta-analysis. BMJ Open. (2021) 11(2):e038385. 10.1136/bmjopen-2020-038385 33622936 PMC7907876

[17] Fyksen TS Seljeflot I Vanberg P Atar D Halvorsen S. Platelet activity, coagulation, and fibrinolysis in long-term users of anabolic-androgenic steroids compared to strength-trained athletes. Thromb Res. (2024) 238:60–6. 10.1016/j.thromres.2024.04.027 38676967

[18] Elbers LPB Fliers E Cannegieter SC. The influence of thyroid function on the coagulation system and its clinical consequences. J Thromb Haemostasis. (2018) 16(4):634–45. 10.1111/jth.13970 29573126

[19] Schwartz J Leber MD Gillis S Giunta A Eldor A Bussel JB. Long term follow-up after splenectomy performed for immune thrombocytopenic purpura (ITP). Am J Hematol. (2003) 72(2):94–8. 10.1002/ajh.10253 12555211

[20] Bollova D Reptova A Valkovicova T Gazdikova K Simkova I. Risk of chronic thromboembolic pulmonary hypertension after splenectomy. Bratisl Med J. (2024) 125(3):176–82. 10.4149/BLL_2024_28 38385544

[21] Jaïs X Ioos V Jardim C Sitbon O Parent F Hamid A Splenectomy and chronic thromboembolic pulmonary hypertension. Thorax. (2005) 60(12):1031–4. 10.1136/thx.2004.038083 16085731 PMC1747270

[22] Kimmig LM Palevsky HI. Review of the association between splenectomy and chronic thromboembolic pulmonary hypertension. Ann Am Thorac Soc. (2016) 13(6):945–54. 10.1513/AnnalsATS.201512-826FR 27058013

[23] Hamed EM Ibrahim ARN Meabed MH Khalaf AM El Demerdash DM Elgendy MO The outcomes and adverse drug patterns of immunomodulators and thrombopoietin receptor agonists in primary immune thrombocytopenia Egyptian patients with hemorrhage comorbidity. Pharmaceuticals. (2023) 16(6):868. 10.3390/ph16060868 37375815 PMC10303883

[24] Ghanima W Gernsheimer T Kuter DJ. How I treat primary ITP in adult patients who are unresponsive to or dependent on corticosteroid treatment. Blood. (2021) 137(20):2736–44. 10.1182/blood.2021010968 33827138

